# Supplementary material for: The Association between BZIP Transcription Factors and Flower Development in Litsea cubeba
Source: Int J Mol Sci. 2023 Nov 23;24(23):16646. doi: 10.3390/ijms242316646 (PMC10705912; doi:10.3390/ijms242316646)
Supplement: Supplementary file 1 [file ijms-24-16646-s001.zip › ijms-2712386-supplementary.pdf]

Supplementary Materials:

Table S1. Characteristics of *LcubZIP* genes. This table presents the gene ID, molecular weight, isoelectric point (pI), and GRAVY (grand average of hydropathy) values for the *LcubZIP* genes.

Gene ID	Molecular Weight	pI (isoelectric point)	GRAVY
LcubZIP1	50471.53	5.41	-0.502
LcubZIP2	36970.74	6.93	-0.818
LcubZIP3	15523.57	8.76	-0.904
LcubZIP4	39247.19	4.62	-0.479
LcubZIP5	50461.95	7.29	-0.615
LcubZIP6	50074.31	7.17	-0.469
LcubZIP7	105755.3	6.41	-0.503
LcubZIP8	36507.01	8.87	-0.853
LcubZIP9	31169.43	8.28	-0.597
LcubZIP10	12198.19	11.53	-0.95
LcubZIP11	62177.45	6.46	-0.902
LcubZIP12	18083.61	10.72	-0.668
LcubZIP13	27745.01	5.47	-0.504
LcubZIP14	54184.37	6.58	-0.417
LcubZIP15	39148.36	7.73	-0.845
LcubZIP16	34798.49	6.63	-0.846
LcubZIP17	18077.3	5.93	-1.226
LcubZIP18	24851.61	5.37	-0.67
LcubZIP19	15821.46	8.76	-0.477
LcubZIP20	39419.47	6.36	-0.837
LcubZIP21	44799.68	6.2	-0.858
LcubZIP22	22528.36	9.35	-0.715
LcubZIP23	77064.99	6.63	-0.478
LcubZIP24	45809.98	5.46	-0.717
LcubZIP25	38978.66	5.69	-0.621
LcubZIP26	41583.33	8.5	-0.46
LcubZIP27	51346.45	8.46	-0.503
LcubZIP28	11142.32	5.27	-1.136
LcubZIP29	32545.32	6.10	-0.636
LcubZIP30	11796.52	10.38	-1.085
LcubZIP31	18520.44	9.56	-1.148
LcubZIP32	44971.83	6.96	-0.729
LcubZIP33	44971.83	6.96	-0.729
LcubZIP34	45122.29	5.36	-0.182
LcubZIP35	17334.62	6.29	-0.668
LcubZIP36	20616.06	6.12	-0.874
LcubZIP37	63410.16	7.74	-0.878
LcubZIP38	61123.8	8.83	-0.932
LcubZIP39	17771.41	11.42	-0.749
LcubZIP40	48292.03	6.66	-0.461
LcubZIP41	37470.39	8.82	-0.994
LcubZIP42	29406.58	6.59	-0.763
LcubZIP43	38401.44	9.24	-0.627

LcubZIP44	22831.12	9.00	-0.438
LcubZIP45	29200.13	9.51	-0.939
LcubZIP46	33721.69	6.16	-0.474
LcubZIP47	35532.31	8.64	-0.567
LcubZIP48	41477.92	6.77	-0.452
LcubZIP49	44263.58	6.03	-0.778
LcubZIP50	23218.17	7.75	-0.709
LcubZIP51	16557.93	6.91	-0.582
LcubZIP52	18448.45	9.36	-1.198
LcubZIP53	36255.97	6.40	-0.485
LcubZIP54	16272.72	6.60	-0.696
LcubZIP55	30096.22	9.11	-0.661
LcubZIP56	31772.42	6.15	-0.721
LcubZIP57	15537.59	8.76	-0.899
LcubZIP58	32351.01	5.57	-0.757
LcubZIP59	34249.38	5.97	-0.763
LcubZIP60	41957.22	6.37	0.739
LcubZIP61	45897.22	8.89	-0.674
LcubZIP62	42760.99	9.29	-0.755
LcubZIP63	41419.24	6.02	-0.488
LcubZIP64	42729.65	6.34	-0.755
LcubZIP65	16131.39	6.83	-0.79
LcubZIP66	45016.11	9.12	-0.693
LcubZIP67	16683.12	6.42	-0.724
LcubZIP68	50565.61	6.37	-0.498

Table S2. Primer Sequences for LcbZIP Genes. The table presents the gene ID along with the corresponding left and right primer sequences for the six candidate LcbZIP genes that potentially regulate stamen or pistil degradation during flower development .

Gene ID	Left Primer	Right Primer
LcbZIP5	TGGCCGTGAAATCAGATGTC	CTGCCGGCGTCTTCCA
LcbZIP18	GGCGTCTTCCAGTTCAGCTA	GCACAGCCGTTAGATTCTCC
LcbZIP20	CTCACTCCCCGACATTCAGA	CGACAAGCTCCTGAAATGGG
LcbZIP22	TTTGCTTCCACTCCTCCCAT	GGGTCGTCTTCTTTTGTCCTG
LcbZIP35	TTCTCCCAGTGGCACTTCTT	TCATCCTTGACCTCCTTGCA
LcbZIP40	CAGCTGATCAACCAAAGCAA	TACAGGCACCAGTTGTGGAA
